# Epidemiology of Cancer-Associated Thrombosis in Asia: A Systematic Review

**DOI:** 10.3389/fcvm.2021.669288

**Published:** 2021-05-21

**Authors:** Lai Heng Lee, Chandramouli Nagarajan, Chuen Wen Tan, Heng Joo Ng

**Affiliations:** Singapore General Hospital, Singapore, Singapore

**Keywords:** cancer, thrombosis, Asia, pulmonary embolism, deep vein thrombosis

## Abstract

The epidemiology of cancer associated thrombosis (CAT) in Asia is less well-studied and differs from that in the western countries. Here, we systematically examine population based and hospital-based studies reported between 1995 and 2020 to understand the epidemiology of CAT in Asia. From population-based studies, the estimated incidence of VTE in cancer patients was 1.85–9.88 per 1,000 person-years. The incidence of CAT in Asia is significantly higher than non-cancer associated VTE in the general population and cancer is perhaps the most important risk factor for VTE. Hospital-based studies were heterogeneous in study designs and reveal a wide range of prevalence of VTE among cancer patients at 0.5–44.6% while the cancer prevalence rates among VTE patients ranged from 6.1 to 65.5%. The cancer sites most associated with VTE and risk factors were similar between Asian and Western studies. CAT has a major impact on the survival of patients with cancer in Asia, but thromboprophylaxis is not commonly practiced and validated risk assessment tools are lacking. This study highlights the urgent need for large multinational epidemiological studies in Asia to establish the true burden of CAT and to guide appropriate prevention strategies.

## Introduction

The association of thromboembolic events with cancer was first described in the nineteenth century (1) and has been irrefutably shown to be a common and detrimental complication of cancers since then (2–4). Patients with active cancers have a four- to seven-fold increased risk of venous thromboembolism (VTE) and account for 20–51% of the overall VTE incidence (5–7). VTE is associated with high morbidity and leads to a two- to six-fold increase in the risk of death (1, 8, 9) in cancer patients. With increased morbidity, mortality and cost of treatment, the rising incidence of cancer-associated thrombosis (CAT) places an increasing burden on the healthcare system (4, 10).

Contrary to early impressions that VTE is uncommon among Asians (11), recent evidence supports a rising incidence in this part of the world, with cancer identified as one of the most common risk factors (12). Concurrently, more convincing epidemiological data have emerged to lend weight to race being a factor in thrombogenicity in VTE with Asians being less predisposed (12, 13). Against these divergent factors, the VTE risk profile of the Asian patient and the actual disease burden of CAT in Asia remains to be ascertained. Published literature on CAT in Asia are mainly country-centric and major multicenter and multinational data are lacking. As current published guidelines on prevention and management of CAT are largely based on data from Caucasian populations, there is, therefore, a credible gap of knowledge that clouds the applicability of these recommendations in Asia.

In this review, we evaluated the epidemiology of CAT in Asia by systematically examining the published literature between 1995 and 2020 and sought to identify gaps in information that will eventually aid the understanding of CAT among Asians to guide prevention and treatment strategies.

## Materials and Methods

### Selection of Studies

A systematic search of the literature was conducted using the PICOT approach (14) and PRISMA checklist to identify publications that reported incidence of CAT, defined as clinically diagnosed VTE—including deep vein thrombosis (DVT) and/or pulmonary embolism (PE) in patients with active cancer, as well as studies that reported the rates of cancer among patients with VTE. Other pre-determined inclusion criteria were: (i) the population studied included at least 90% Asian participants or the study was conducted in one of the specified Asian regions (Afghanistan, Bangladesh, China including Hong Kong and Macau, India, Indonesia, Japan, Korea, Malaysia, Mongolia, Pakistan, Philippines, Singapore, Sri Lanka, Taiwan, Thailand, Vietnam); (ii) the full publication was available in English; (iii) the reported study included at least 100 subjects from the Asian population of interest; (iv) the publication reported the risk factors, demographic data and disease burden associated with CAT; (v) the publication date was from January 1995 to January 2020.

**Figure 1:**
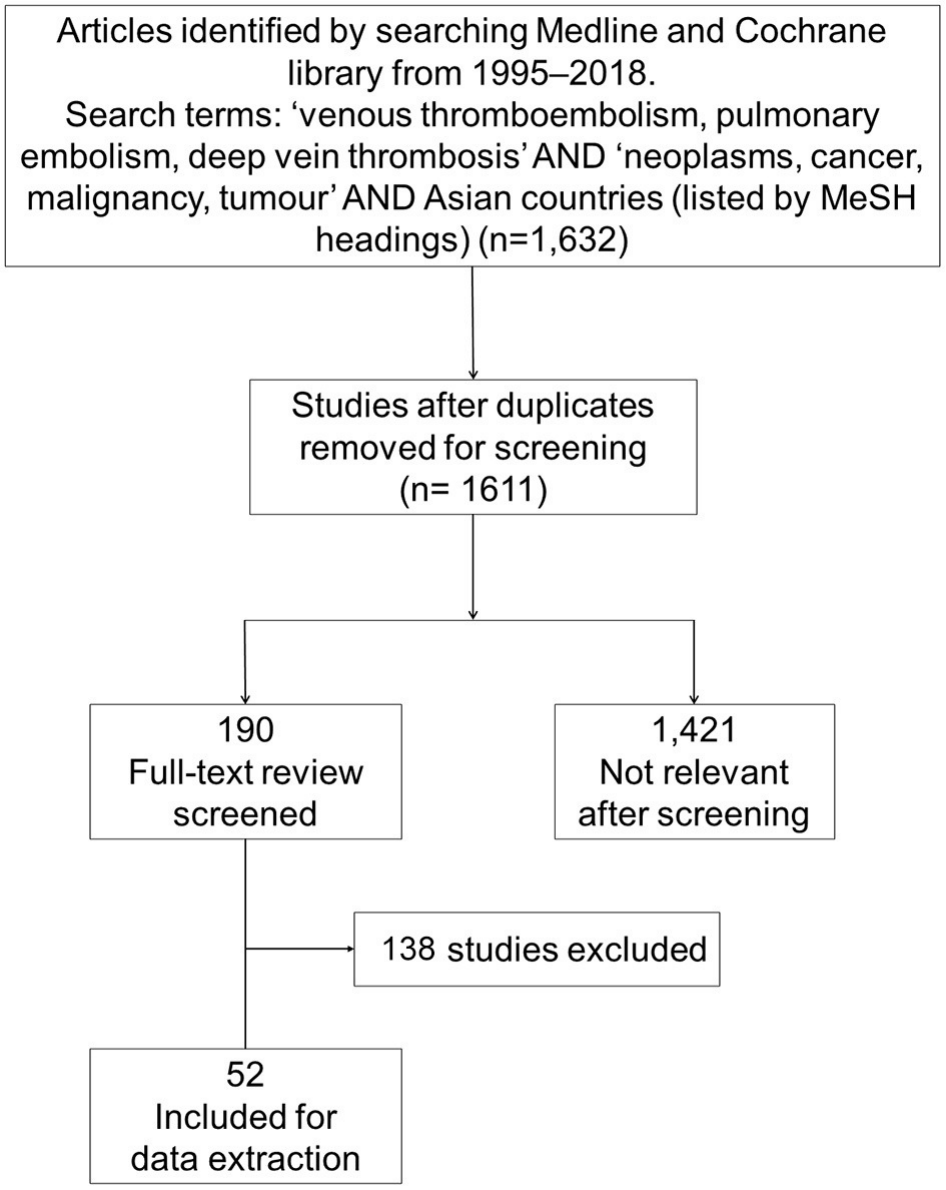
PRISMA flowchart of included studies.

A search string was developed and the final literature search was conducted in PubMed (MEDLINE) and the Cochrane Library on 30 January 2019. Duplicates were removed and the title and abstract for each manuscript were independently screened by two reviewers against the pre-defined inclusion and exclusion criteria (Figure 1). Full-text manuscripts were obtained for all abstracts that were deemed potentially eligible. Two reviewers independently screened the full-text articles for eligibility using a standardized screening form. Discrepancies were resolved by discussion with a third reviewer. The reference lists of all papers included were hand-searched to identify additional manuscripts that may have been omitted in the initial search; the “related article” feature in PubMed was also used to identify additional articles. Google Scholar, EMBASE and grey literature were also searched to cross-check for additional relevant articles (Figure 1).

### Data Collection and Analysis

Studies were grouped, according to the study protocol, as belonging to one of the following categories: (i) Population-based studies, defined as population-based estimates of CAT incidence in the general population, from sources such as national health system registries or health insurance databases; (ii) Hospital-based studies, defined as single-institution or multicenter estimates of CAT incidence in the general population, or reports of CAT rates in consecutive hospitalized patients, surgical patients or oncology patients; (iii) Studies that evaluated CAT risk scoring; (iv) Impact studies, defined as reports of CAT-associated mortality in the general population or in respective cancer subtypes, or estimates of CAT-associated healthcare costs; and (v) CAT treatment, defined as studies that reported prophylaxis or treatment outcomes for CAT. As mentioned, the main focus of this manuscript is CAT epidemiology; however, important aspects on treatment were gleaned from the reviewed literature, which were then included.

Studies were further subcategorized based on whether they reported CAT for patients with any cancer types or those with specific cancers. Individual studies may be used in more than one subcategory; hence, the sum of the number of studies per topic may be more than the total number of studies included.

The following information was extracted using a standardized data extraction form: country, study period, study design and cohort, population size, cancer type, number and incidence of VTE, DVT and PE, and patient mortality (overall and VTE-related). Results were then summarized descriptively.

## Results

The search strategy identified 1,611 papers after removing duplicates. Initial title and abstract searches identified 190 potentially relevant studies. After screening, 52 studies met the eligibility criteria for data extraction (Figure 1). Nine studies were population-based studies and 43 were hospital-based studies from China, Hong Kong, India, Japan, Korea, Singapore, Taiwan, and Thailand.

### Population-Based Studies of CAT

There is a paucity of studies reporting population-based estimates of CAT incidence.

#### VTE in Cancer Patients of all Cancer Types

Using data from their National Health Insurance Research Database (NHIRD), two retrospective studies from Taiwan were the only ones available that examined population-based estimates of VTE incidence in cancer patients. The first study, conducted between 1997 and 2005, included 497,180 newly diagnosed cancer patients with a median follow-up duration of 21.3 months. It found an estimated incidence of VTE of 1.85 per 1,000 person-years (15), with a clear rising trend over the nine study years. VTE was concomitantly diagnosed with cancer in 28.5% of patients. The sites of VTE were not elaborated. The median time to VTE after cancer diagnosis was 5.8 months, with the highest incidence of VTE seen in the first year after a cancer diagnosis. Thereafter, the incidence of VTE decreased time-dependently in the following years (*p* < 0.001). Risk factors identified for developing VTE included female gender over the age of 40 years, a previous history of VTE, and certain cancer types. The rates and risks of VTE were highest in patients with prostate, lung and gynecological cancers, renal carcinoma and myeloma, with odds ratio (OR) between 1.367 to 1.797 on univariate analysis and 1.269 to 1.626 on multivariate analysis with logistic regression. Pancreatic cancer was associated a lower VTE rate with an OR of 0.887. The median overall survival of patients with DVT and PE was 32.5 and 31.2 months, respectively, compared with 70.5 months in patients without VTE.

The later study identified 43,855 hospitalized patients with newly diagnosed cancer between 2001 and 2008 (16). Patients with CAT were identified using two algorithms: the first using VTE disease codes alone, and the second using VTE disease codes plus any treatment for VTE. The number of VTE cases with these two algorithms were 1,388 and 473, respectively, for incidence rates of 9.88 and 3.35 per 1,000 person-years. The distribution of VTE sites, based on the two algorithms, were as follows: PE (8.5%/16.1%); PE and DVT (1.1%/3.2%); extremities (5.3%/4.0%); vena cava (2.7%/2.5%); renal vein, hepatic vein or portal vein (52.9%/19.0%); unspecified site (27.7%/53.9%); and multiple sites (1.5%/1.3%). Based on disease coding, the cancer types with the highest incidence rates of VTE were cancers of the liver (68.2 per 1,000 person-years), pancreas (27.8 per 1,000 person-years), lung (17.2 per 1,000 person-years), multiple myeloma (10.6 per 1,000 person-years) and non-Hodgkin's lymphoma (9.32 per 1,000 person-years), which together accounted for 67.6% of the VTE cases. Other risk factors for VTE among cancer patients included prior history of VTE [odds ratio (OR) 4.3; 95% confidence interval (CI) 1.6, 11.7] and comorbid diseases such as hypertension, arterial embolism, obesity, rheumatologic diseases, surgical interventions and chemotherapy (OR 3.6; 95% CI 3.0, 4.4). Long-term anticoagulation was initiated in 64.1% of patients [46.3% received warfarin alone while 9.6% received low-molecular-weight heparin (LMWH)]. The majority of patients were treated for a relatively short duration, with 58.7% receiving anticoagulation for ≤ 3months while only 9.4% received treatment for ≥12months.

#### VTE in Cancer Patients of Specific Cancer Types

There was only one study that discussed the VTE risk in patients with specific cancer types.

Tsai et al. (17), in a nationwide population-based study in Taiwan (2003–2008), evaluated the 5-year incidence of VTE in 1013 patients with cervical cancer. The cumulative risk for VTE was significantly higher in cervical cancer patients than in controls (patients who underwent appendectomy) (3.3 vs. 0.3%, *p* < 0.001) (17). The VTE sites were not reported. Mortality rates were significantly higher in patients with VTE (survival rate 30.3% in the VTE group vs. 75.3% in patients without VTE, *p* < 0.001).

#### Cancer as a Risk Factor for VTE and Impact on Outcomes

Four studies addressed cancer as a risk factor for VTE and its impact on outcomes in population databases.

An observational cohort study of 5,347 adult patients in Taiwan with a discharge diagnosis of VTE from 1 January 2001 to 31 December 2002 found that malignant neoplasms were present in 1,123 patients (21.6%) and were only second to major surgery (38%) as the most common risk factor for VTE (18). The commonest malignant neoplasms present were gastrointestinal and hepatic (34.1%), urogenital (29.3%) and respiratory tract (24.0%) cancers. Malignancy-associated VTE had a higher rate of recurrence, [adjusted odds ratio (OR) 1.64, 95% CI 1.26–1.99] and is an independent predictor of increased risk of 30-day mortality [hazard ratio (HR) 2.28, 95% CI 1.81–2.87, *p* < 0.001].

In South Korea, an observational study of 808 patients diagnosed with acute pulmonary thromboembolism (APTE) from January 1998 and December 2000 found that cancer was present in 15.8% of these patients. The most common sites of cancer were lung, gastrointestinal tract, and liver. Multivariate logistic regression identified lung cancer as an independent risk factor of mortality (OR 9.2, 95% CI 1.96–43.27, *p* = 0.03) (19).

In Singapore, a cohort study of 130 cases of fatal PE on post-mortems (1989–1993) found that 11% of fatal PE cases had cancer (20, 21); 88% of PE arose from deep veins of the legs. PE was unsuspected in 75% of fatal cases.

A retrospective analysis of cancer-associated VTE in patients with advanced solid cancers using data from the Korean VTE registry showed that the 6- and 12-month cumulative incidences of recurrent VTE in cancer patients were 20.6 and 27.0%, respectively. Recurrences within 3 months after index VTE event presented as Isolated PE (51.0%), isolated VTE (28%) and PE + DVT (66%) (22). Pancreas as the primary tumor site, poor performance status and initial presentation with PE are independent risk factors for recurrent VTE in patients with advanced solid tumors receiving anticoagulation therapy after index VTE. Patients with recurrent VTE had significantly shorter overall survival than those without (median OS 8.4 vs. 13.0 months, *p* = 0.001).

#### Subsequent Cancer Risk Following VTE

Only one study explored the subsequent cancer risk of patients following VTE. A population-based cohort study (*n* = 27,751) of patients diagnosed with unprovoked VTE between January 1998 and December 2008 showed that subsequent cancer risk was significantly higher in patients with unprovoked VTE than in the variable-matched control group (adjusted HR 2.26; 95% CI 2.16, 2.37) (23). This increased risk was for all site-specific cancer sites. Patients aged 50 to 62 years in the VTE group had a 1.2-fold increased risk of cancer compared with the control cohort (*p* < 0.05) after 3 years of follow-up. Compared with female patients in the non-VTE group, female VTE patients in this age-group also had a higher risk of cancer (*p* < 0.05). Patients in the VTE group with cancer were associated with a higher 1-year mortality risk compared with patients in the non-VTE group with cancer (adjusted OR 2.18; 95% CI 1.98, 2.4, *p* < 0.001).

### Hospital-Based Studies of CAT

#### Rates of VTE in Cancer Patients

Hospital-based studies that examined the rates of VTE in cancer patients are summarised in Table 1. The included studies were heterogeneous in terms of study design and patient types (cancer type). The reported prevalence of VTE among cancer patients in these studies varied widely, ranging from 0.5 to 44.6%. The large-scale studies (≥1,000 included patients) reported rates that ranged from 0.5 to 19.3% compared with the studies with <1,000 patients, which ranged from 1.3 to 44.6%. Majority of studies only evaluated lower limb DVT and/or PE specifically. Six studies reported all VTEs but not all clearly specify the sites (30–33, 35, 36). Of these, three studies reported VTE at unusual sites ~10–50% (31, 33, 36). Only one study reported 75% of VTE at unusual sites in greater details but it was in the context of myeloproliferative neoplasms (42).

**Table 1:** VTE rates in studies involving cancer patients.

**Country (study period), references**	**Study design and cohort (including cancer type) (total number of patients)**	**Number (%) of patients (VTE/DVT/PE/DVT** **+** **PE)**				**Risk factors identified**	**Mortality (n, %)**	**Anticoagulation (n, % of VTE patients)**
		**VTE**	**DVT**	**PE**	**DVT + PE**			
**Studies on all cancer types**								
**Large-scale studies (population size** **≥1,000)**								
India (2011–2014)Nair et al. (24)	Retrospective review of medical records of patients with newly diagnosed solid tumours (lung, ovary, stomach, breast, rectal/colon) (11,796)	58 (0.5) (only lower limb DVT and PE reported)	53^a^ (0.4)	5 (<0.1)	0	NR	NR	Prophylaxis: 1 (1.7)Treatment: NR
**Small-scale studies (population size** ** <1,000)**								
Japan (2003–2010)Yamashita et al. (25)	Retrospective review of medical records of patients with cancer (hematopoietic tumours, hepatocellular carcinoma, lung, stomach, uterus) (478^b^)	NR	NR	NR	62 (13.0) (only lower limb DVT and PE only)	NR	VTE-related: NR 90-day all-cause: 7 (9.7) with VTE/27 (8.5) without VTE	NR
**Studies on specific cancer types**								
**Large-scale studies (population size** **≥1,000)**								
China (2015–2017)Xiong et al. (26)	Prospective multi-centred case-control study of lung cancer patients (small cell lung cancer, NSCLC) (9,527)	1,841 (19.3) (only lower limb DVT and PE reported	825 (8.7)	560 (5.9)	456 (4.9)	NR	VTE-related: NR 30-day all-cause: 104 (10.2) among PE cases	NR
Korea (2003–2009)Sun et al. (27)	Retrospective review of medical records of lung cancer patients (NSCLC, SCLC) (8,014)	NR/NR/180 (2.2)/NR (only PE reported)				Advanced stageHistory of chemotherapy	PE-related: 6 (3.3) All-cause: 115 (63.9)	Prophylaxis: NRTreatment: 51 (45.1)
Korea (2005–2014)Cha et al. (28)	Retrospective review of medical records of patients with lung cancer (adenoCA, SCC, SCLC, large cell carcinoma, NSCLC, not otherwise specified) (5,005)	NR	NR	267 (5.3) 27 at cancer diagnosis; 240 after diagnosis	55 (1.1)	NR	PE-related: 2 (0.7) In-hospital all-cause: 20 (7.5)	Prophylaxis: 220 (82.4)Treatment: NR
China (2004–2013)Wang et al. (29)	Prospective cohort study of NSCLC patients (adenoCA, sarcomatoid carcinoma, SCC) (4,726)	61 (1.3) (only lower limb DVT and PE reported)	45 (9.5)	5 (0.1)	11 (2.3)	Serous effusionFever Increased leucocyte counts Hyponatremia Increased alanine aminotransferase level	PE-related: 1 (1.6) All-cause: NR	Prophylaxis: NRTreatment: 58 (95.1)
Korea (2003–2008)Lee et al. (30)	Review of prospective databases of gastric cancer patients (2,085)	73 (3.8) (all VTEs but specific sites not reported)	NR	NR	NR	Older ageNo surgeryHigher cancer stage	NR	Prophylaxis: NRTreatment:Any anticoagulant: 28 (38)LMWH: 20 (27)Warfarin: 8 (11)
Korea (2003–2009)Choi et al. (31)	Retrospective review of medical records of patients with colorectal cancer (rectum, left, and right colon) (2006)	91 (4.5) (mixed but no clear reporting of unusual site VTE but <50%; at most 45/91)	58 (2.9)^c^	33 (1.6)	NR	NR	NR	Peri-operative prophylaxis: 200 (11.8), all heparin Treatment: NR
Korea (2006–2010)Lee et al. (32)	Retrospective review of medical records of NSCLC patients (SCC, adenoCA, others) (1,998)	131 (6.6) (VTE site not specified)	NR	NR	NR	Advanced agePneumonectomyPalliative radiotherapyIneligibility for surgerySmoking	NR	Prophylaxis: NR Treatment:Any: 119 (90.8) (74.2% in asymptomatic vs 96.0% in symptomatic).Warfarin: 16 (12.2)LMWH then warfarin: 17 (13.0)LMWH: 86 (65.6)
Korea (2008–2014)Go et al. (33)	Retrospective review of medical records of lung cancer patients (adenoCA, SCLC, SCC, others) with VTE treated with therapeutic anticoagulation (1,707)	134 (7.9) (not all VTE sites are specified)	NR	102 (6.0)	NR	NR	NR	Prophylaxis: NRTreatment:Any: 134 (100) as part of inclusion criteriaWarfarin: 111 (82.9)LMWH: 22 (16.4)Rivaroxaban: 1 (0.7)
China (2012–2015)Shen et al. (34)	Retrospective review of medical records of non-small cell lung cancer patients (1,560)	32 (2.0) (only lower limb DVT and PE reported)	14 (0.9)	12 (0.8)	6 (0.4)	Weight lossPoor performance statusIncreased c-reactive proteinProlonged prothrombin time	NR	Prophylaxis: NRTreatment: 29 (90.6), all with warfarin and LMWH
**Small-scale studies (population size** ** <1,000)**								
Korea (2007–2011)Lee et al. (35)	Retrospective review of medical records of AML patients (*de novo* leukaemia, AML with myelodysplastic changes, therapy-related myeloid leukaemia) (811)	26 (3.1) (VTE sites not specified)	NR	NR	NR	Advanced ageHigh cytogenetic risk	VTE-related: NR All-cause: 325 (40.1)	NR
Japan (2007–2016)Kanaji et al. (36)	Retrospective review of medical records of lung cancer patients (SCLC, adenoCA, SCC, others, NSCLC, not otherwise specified) (716)	16 (2.2)	8 (1.1)	2 (0.3)	4 (0.6)	NR	NR	NR
China (2009–2011)Zhang et al. (37)	Prospective cohort study of newly diagnosed lung cancer patients (adenoCA, SCC, SCLC, others) (698)	89 (13.2) (only lower limb DVT and PE reported)	42 (6.0)^d^	33 (4.7)	14 (2.0)	DVT: distant metastasis, leucocytosisPE: adenoCA, anaemia	No deaths reported	Prophylaxis: NRTreatment: 89 (100)
Korea (2008–2010)Park et al. (38)	Prospective cohort study of patients with lymphoma (B/T cell lymphoma, aggressive B cell lymphoma, peripheral T cell lymphoma, central nervous system lymphoma, Hodgkin lymphoma) (686)	54 (7.9) (only lower limb DVT and PE reported)	33 (4.8)^e^	0	21 (3.1)	ChemotherapyPrimary CNS lymphomaOlder age	No deaths	Prophylaxis: 0 (0)Treatment: 54 (100)
China (2012–2017)Dou et al. (39)	Prospective cohort study of newly diagnosed NSCLC patients (adenoCA, SCC, other NSCLC) (605)	71 (11.7) (only lower limb DVT and PE reported)	44 (7.3)^f^	7 (1.2)	20 (3.3)	AdenoCApoor performance statusEGFR wild-type	PE-related: 3 (4.2) All-cause: NR	NR
China (2014–2016)Liu et al. (40)	Retrospective review of medical records of patients with gynaecologic malignancies (cervical, ovarian, endometrial, broad ligament) (376)	39 (10.3) (only lower limb DVT and PE reported)	36 (9.6)	1 (0.3)	2 (0.5)	Thromboelastography coagulation index valueD-dimerArrhythmiaCoronary heart diseaseSurgery within 4 weeksChemotherapy within 4 weeks	NR	NR
China (2013–2014)Fei et al. (41)	Prospective cohort study of patients with NSCLC (adenoCA, SCC, others NSCLC) (205^g^)	46 (22.4) (only lower limb DVT reported)	46 (22.4)	NR	NR	NR	VTE-related: NR All-cause: 36 (78.3) with VTE/24 (15.1) without VTE	NR
Thailand (2003–2013)Duangnapasatit et al. (42)	Retrospective review of patients with myeloproliferative neoplasms (essential thrombocythemia, polycythemia vera) (157)	8 (5.1) (all sites reported)	1 (0.6)	1 (0.6)	0	NR	No deaths	NR
Japan (2006–2012)Yokoyama et al. (43)	Retrospective review of medical records of patients with diffuse large B-cell lymphoma (142)	15 (10.6) (only lower limb DVT and PE reported)	13 (9.2)	0	2 (1.4)	Poor performance status	No deaths	Prophylactic VKA: 1 (6.7)Treatment, any: 11 (73.3)Treatment, UFH: 8 (53.3)Treatment VKA: 3 (20.0)

a *48 cases (83%) were lower limb DVT*.

b *Cancer patients only; control group included 121 patients without cancer*.

c *Includes DVT of the lower extremity plus intraabdominal venous thrombosis*.

d *All lower limb*.

e *14 (42%) patients in neck/upper limb; 12 (36%) in lower limb; 7 (22%) in the abdomen/pelvis*.

f *10 (23%) in the upper extremity/neck; 32 (73%) in the lower extremity/pelvis; 2 (4%) in the upper and lower extremities*.

g *Cancer patients only; control group included 102 patients without cancer*.

*AdenoCA, adenocarcinoma; AML, acute myeloid leukaemia; DVT, deep vein thrombosis; UFH, unfractionated heparin; LMWH, low molecular weight heparin; NR, not reported; NSCLC, non-small cell lung cancer; PE, pulmonary embolism; SCC, squamous cell carcinoma; SCLC, small cell lung cancer; VKA, vitamin K antagonist; VTE, venous thromboembolism*.

None of the hospital-based studies on all cancer types reported risk factors other than cancers for CAT. On the other hand, 11 studies on specific cancer type reported risk factors for CAT, as described in Table 1.

Four studies reported VTE-related deaths (mostly associated with PE). The three large-scale studies with VTE-related deaths reported rates ranging from 0.7–3.3% (27–29) whereas one small-scale prospective cohort study of newly diagnosed NSCLC patients reported a 4.2% VTE-related mortality rate (39). Three large-scale studies reported all-cause mortality rates from 7.5 to 63.9% (26, 28, 31) while the rates reported by three small-scale studies were from 9.7 to 78.3% (25, 35, 41).

Although the main focus of this manuscript is CAT epidemiology, important aspects on treatment were gleaned from the reviewed literature. Nine studies reported rates of VTE treatment using anticoagulants. The reported treatment rates ranged from 45.1 to 100% (27, 29, 30, 32–34, 37, 38). Of these, four studies did not evaluate the details of treatment while five studies reported the anticoagulant used. Among the five studies that specified the anticoagulant, four studies reported a higher rate of LMWH use. The only study with high warfarin use was a Korean retrospective review on lung cancer patients seen from 2008 to 2014 (33). Five studies of varying study populations and designs reported rates of prophylactic anticoagulation; these rates ranged widely from 0 to 82.4% (24, 28, 31, 38, 43). No study specifically assessed the complications of anticoagulation in terms of bleeding complications and recurrence rates while on treatment.

#### Rates of Cancer in VTE Patients

Hospital-based studies that examined the rates of cancer among VTE patients are summarised in Table 2. The included studies were heterogeneous in terms of study design and patient types (VTE type and method of VTE detection). The studies also reported a wide range of cancer prevalence rates (from 6.1 to 65.5%) and cancer-related findings were often not thoroughly described or discussed in these studies (44–57, 59, 60, 62). Two studies analysed the mortality rates of VTE patients according to cancer status. Yokoi et al. (58) reported an HR for mortality among those with vs. without cancer of 12.5 (*p* < 0.001) by univariate analysis, and 9.1 (*p* = 0.003) by multivariate analysis (58). On the other hand, Lee et al. (61) reported that active malignancy and/or chemotherapy were associated with an increased risk of 30-day all-cause mortality (OR, 28.87; 95% CI, 6.564, 126.950; *p* = 0.001) by multivariate analysis. Among these studies, only one study reported the treatment of VTE among patients with cancer. Of the 120 VTE patients with malignancy, 100 patients (83.3%) received an anticoagulant, including 90 (75.0%) with warfarin and 16 (13.3%) with direct oral anticoagulants (13.3%) (58).

**Table 2:** Cancer rates in studies involving VTE patients.

**Country (study period), references**	**Study design and cohort**	**Number (%) of patients**			**Mortality (% of VTE)**
		**Total**	**VTE (n, % of total)**	**Cancer in VTE (n, % of VTE) and cancer types**	
**Large-scale studies (population size** **≥1,000)**					
China (2007–2016)Zhang et al. (44)	Retrospective review of discharge records from 90 hospitals	37,106,474	105,723 (0.3)	13,616 (12.9) with unspecified cancer	NR
Singapore (2002–2003)Ng et al. (45)	Prospective recruitment of patients diagnosed with DVT	109,217	495 (0.5)	115 (23.2) with unspecified cancer	PE-related: 6 (1.2) All-cause: 61 (12.3)
Thailand (2007–2008)Aniwan et al. (46)	Prospective recruitment of all patients admitted for more than 3 days	7,126	42 (0.6)^a^	22 (52.4) with unspecified cancer	PE-related: 9 (21.4) All-cause: 21 (50.0)
Japan (2008–2013)Nakamura et al. (47)	Retrospective review of medical records diagnosed with VTE	3,578^b^	3554^b^	896 (25.2) with unspecified cancer	NR
Hong Kong (2004–2015)Huang et al. (48)	Observational study (hospital VTE registry)	2,214	2,214 (100)	1,096 (49.5) with unspecified cancer	NR
Thailand (2009)Rojnuckarin et al. (49)	Prospective recruitment of patients admitted to internal medicine wards at high-risk of VTE	1,290	27 (2.1)	13 (48.1) with solid tumours (adenocarcinoma; lymphomas)	PE-related: NR All-cause: 6/20^c^ (30.0)
China (2004–2013)Wang et al. (50)	Retrospective review of medical records of patients with VTE	1,048	1,048 (100)	109 (10.4) at cancer of the lung, liver, uterus, bowel; lymphoma	NR
**Small-scale studies (population size** ** <1,000)**					
Singapore (1998–2001)Tan et al. (51)	Retrospective review of medical records of symptomatic patients referred for DVT ultrasound	862	277 (32.1) with DVT	46 (16.6) with unspecified cancer	NR
China (2004–2015)Chen et al. (52)	Retrospective review of medical records of vascular surgery patients with DVT	783	783 (100)	55 (7.0) with cancer of the stomach, lung, cervix, breast, ovary; myeloproliferative disorders	NR
China (2009–2013)Zhang et al. (53)	Cohort study of acute PE patients	578^d^	563^d^ (97.4)	70 (12.4) with unspecified cancer	PE-related: NR 3-month all-cause: 19/539^d^ (3.5)
Hong Kong (1997–2000)Liu et al. (54)	Retrospective review of medical records of patients with VTE	376	376 (100)	62 (16.5) with cancer of the colon, ovary, cervix; bronchogenic carcinoma; metastatic cancer of unknown origin	NR
Singapore (2008–2013)Mok et al. (55)	Retrospective review of records of patients admitted with acute PE	343	343 (100)	21 (6.1) with unspecified cancer	In-hospital all-cause: 17 (5.0)
Hong Kong (1994–1998)Lee et al. (56)	Retrospective review of medical records of patients who underwent lower extremity duplex venous scans	313	63^e^ (20.1)	10 (15.9) with cancer of the bladder, rectum, sigmoid colon, stomach, liver; leiomyosarcoma	NR
Hong Kong (1999–2012)Chung et al. (57)	Retrospective review of medical records of patients undergoing upper extremity venous sonography examinations	213	29^f^ (13.6)	19 (65.5) with unspecified cancer	NR
Japan (2012–2017)Yokoi et al. (58)	Retrospective review of medical records for *de novo* acute DVT patients	211	211 (100)	120 (56.8) with gynaecological, gastrointestinal, urinary, respiratory, brain cancer	With cancer: 28 (23.3)^g^ Without cancer: 2 (2.2)^g^
Japan (2002–2007)Yamaki et al. (59)	Prospective recruitment of patients diagnosed with cardiopulmonary stable PE evaluated for DVT	203	203 (100)	30 (14.8) with unspecified cancer	PE-related: 5 (2.5) All-cause: 23 (11.3)
China (2010–2014)Deng et al. (60)	Prospective cohort study of PE patients treated in the emergency department	149	149 (100)	12 (8.1) with unspecified cancer	PE-related: NR All-cause: 11 (7.4)
Korea (2013–2015)Lee et al. (61)	Retrospective review of medical records of patients with PE	141	141 (100)	39 (27.7) with unspecified cancer	With cancer: 18/39 (46.2) Without cancer: 10/102 (9.8)
China (2008–2012)Yu et al. (62)	Retrospective review of medical records of patients with idiopathic DVT	128	128 (100)	16 (12.5) with lung, stomach, liver, prostate, or ovarian cancers; cholangiocarcinoma, non-Hodgkin's lymphoma, or metastatic carcinoma of unknown origin	PE-related: 2 (1.6) All-cause: 5 (3.9)
Thailand (NR)Angchaisuksiri et al. (63)	Prospective recruitment of patients referred for hypercoagulable state workup	105	105 (100)	20 (19.0) with gynaecological, lung, gastrointestinal, breast cancer; non-Hodgkin lymphoma	NR

a *19 had DVT, 19 had PE, 4 had both*.

b *Among 3,578 patients with VTE, 3,554 were included in the analysis*.

c *Only for the 20 patients with in-hospital VTE*.

d *Among 578 consecutive patients who were diagnosed with acute PE, 563 were included in the analysis and 539 completed the 3-month follow-up*.

e *53 had DVT, 10 had DVT with PE*.

f *Upper extremity DVT*.

g *3-year all-cause mortality rate*.

*DVT, deep vein thrombosis; NR, not reported; PE, pulmonary embolism; VTE, venous thromboembolism*.

### Risk Scores for Prediction of VTE Occurrence and Recurrence in Cancer Patients

We found four studies addressing risk scores for predicting VTE in cancer patients: three studies on VTE occurrence and one study on VTE recurrence.

The first study on risk scoring for VTE occurrence, by Song et al. (64), evaluated the VTE rates in 262 Chinese patients, of which 147 had lung cancer who underwent surgery without peri-operative thromboprophylaxis. The VTE rate in lung cancer (15%) was twice that of benign cases (7%) (64). Applying the Caprini risk assessment model (RAM), the overall incidence rates of VTE in low-, moderate-, and high-risk groups for VTE were 0%, 12.3% (22/179), and 40.0% (8/20), respectively (*p* < 0.05). Among patients with lung cancer, 98% fell into the moderate- and high-risk groups; VTE incidences were 0% (0/3) in the low-risk, 12.0% (15/125) in the moderate-risk and 36.8% (7/19) in the high-risk groups (*p* < 0.05). This study clearly highlights the predictive effectiveness of the Caprini RAM.

The second was a small study from a single centre in South Korea, which analysed 140 hospitalized patients with active malignancies, for which 31 were hematologic and 109 were non-haematologic malignancies. Participants were examined for DVT by duplex and colour Doppler ultrasonography (DUS) of both legs between days 5 and 14 of their hospital stay. The incidence of VTE by day 14 was 7.1%, including six proximal and four distal DVT cases (65). The VTE rate in this study was low compared with the placebo groups of the Samama et al. (66) and Cohen et al. (67) studies, with VTE rates of 14.9 and 10.5%, respectively. The Padua Risk Prediction score (68) for VTE was applied to each patient but it was modified using the body mass index (BMI) in Asians, for which the cut-off for obesity was adjusted from 30 to 25 kg/m^2^ based on the World Health Organization recommendations (69). Significant findings of being female, having a modified Padua Prediction Score of ≥6 and being hospitalized for ≥13 days were risk factors of VTE in a univariate analysis. The incidence rates of VTE were 2.3, 7.3, and 41.7%, respectively, in patients with 0–1, 2 and 3 of the risk factors of the Padua Prediction Score (65). Bleeding risk was assessed in the 10 patients with VTE using the IMPROVE bleeding risk score and yielded a median score of 5 (range 3.5–10), and three patients (30%) had a score of ≥7.0, which, according to the IMPROVE bleeding risk score scale, indicated a 12% risk of bleeding complications. Three of 10 patients with VTE received relatively short anticoagulation treatment and five patients did not receive any anticoagulation treatment at all. The reasons for not receiving anticoagulation treatment included bleeding risks, poor functional status and reduced life span from cancer.

Finally, Yu et al. (15) developed a risk-stratification scoring system that included age, gender, previous history of VTE and cancer subtypes then validated this using data from the National Health Insurance Research Database of Taiwan. Relative risks associated with VTE for these clinical variables were analysed by univariate and multivariate logistic regression models. A risk score was assigned to each independent value based on the OR in the multivariate analysis. The patients in the developmental cohort were categorised into four discrete risk groups using this scoring. The incidence rates of VTE were 0.5, 0.9, 1.5, and 8.7%, in the very low-risk, low-risk, intermediate-risk, and high-risk groups, respectively, and each group was significantly different from each other (*p* < 0.001) (15). The risk model was then tested in the validation cohort and yielded similar results as the developmental cohort, with incidence rates of VTE at 0.4, 0.9, 1.4, and 9.4% in each risk group, respectively (*p* < 0.001). This scoring system effectively predicted the risk for VTE. Using the very low-risk group as reference; the ORs for the low-risk, intermediate-risk and high-risk groups were 2.2 (95% CI 1.7, 2.3), 3.2 (95% CI 2.8, 3.8), and 23.3 (95% CI 17.8, 30.7), respectively (all *p* < 0.001). When the high-risk score (more than 3) was used as a cut-off point, low sensitivity and positive predictive value (8.7%) were seen (2.8 and 8.7%, respectively, in the development cohort, and 4.1 and 9.4%, respectively, in the validation cohort). However, a high specificity (99.7%) and negative predictive value (NPV) (98.9%) were seen in the development cohort, with similar results observed in the validation cohort (99.6% sensitivity, 99.0% NPV).

The only study on risk scoring for VTE recurrence was a retrospective analysis to validate the Ottawa score for recurrent VTE in a cohort of 546 Korean patients with newly diagnosed VTE (70). The Ottawa score showed a 66% sensitivity, 50% specificity, 22% positive predictive value and 87% negative predictive value in this validation study. The total recurrent VTE rate was 18.1%, which is about twice the rate of recurrences in the derivation study. The Ottawa score was less discriminatory in the validation cohort compared to the derivation study. Using the Ottawa score, Korean patients in the low-risk group (score ≤0) had a 13.2% recurrence rate and the high-risk group (score ≥1) had a 22.4 % recurrence rate; in the derivation study, the respective rates were ≤4.5 and ≥19%. The significantly more gastrointestinal cancers and predominance of vitamin K antagonists for anticoagulant therapy in Korean patients are possible reasons for the differences between the validation and derivation groups.

## Discussion

### Rates of CAT

The reported rates of CAT in Asia vary significantly across studies although this is not surprising given the variability of the studies available. The study designs (prospective vs. retrospective and single center vs. multicenter), circumstances around the diagnosis of CAT (incidental VTE vs. clinically apparent VTE), patient cohorts (inpatient vs. outpatient, surgical vs. medical), study periods, duration of follow up and types and stage of cancer are all crucial factors and variables that could influence the reported CAT rates considerably. Therefore, pooling of data is rendered impossible and even a comparison of findings across studies can sometimes be misleading. Nonetheless, there are still some interesting observations to be made.

Our review showed that the incidence of CAT in all cancer types in Asia, based on population studies, ranged from 1.85 per 1,000 person-years from the data based on insurance claims, to 9.88 per 1,000-years based on hospitalized patients with newly diagnosed cancer. Consistent with the established understanding of hypercoagulability in cancer, these rates are 2.2–11.5 times higher than the rates in the general population (15, 16). The wide range of these reported VTE rates are expected, given that cancer patients who are hospitalized are expected to have substantially increased risks of VTE compared with non-hospitalized patients. Other factors that may contribute to variability in the reported CAT incidences include differences in the type and stages of cancer, the presence of active cancer and the cancer treatment administered (71).

The Asian figures we report here seem to be substantially lower than those reported in Western populations. A population study in the UK reported that the incidence rate of first VTE in patients with active cancer was 58 (95% CI 57–60) per 1,000 person-years (72). Nonetheless, it should be highlighted that the Asian data presented here is derived solely from two retrospective Taiwanese studies based on a national health insurance database. Hence, these findings might not be representative of the true CAT epidemiology across Asia due to differences in ethnicity, cancer epidemiology and healthcare facilities. Furthermore, both studies are retrospective analysis and underreporting and missed cases are possible, resulting in lower incidence rates.

The post-mortem study in Singapore showed that 75% of fatal PE was not suspected ante-mortem (20, 21). Such observations from autopsy studies had suggested that incidence rates of the most serious complication of VTE, fatal PE, could be underestimated in population studies. Large prospective multinational epidemiological studies are conspicuously missing in Asia, rendering the true incidence of CAT uncertain.

Of significance, Yu et al. (15) highlighted that a trend of progressively increasing incidence of VTE over the 9-year study period was observed. Consistently, comparing retrospective hospital series of CAT in lung cancers, a trend of increasing proportion of patients developing VTE in more recent studies can also be observed. This is in keeping with the rising incidence of VTE in population-based studies in Asia (12) and cancer being recognized as a major risk factor for VTE amongst Asian patients. This rise could be attributed to increased awareness of CAT and VTE in general in Asia as well as the aging demographics associated with other thrombotic risk factors.

Even though the observed rates of CAT in Asia appeared to be lower than other populations, cancer remains as one of the most important risk factors for VTE. In Taiwan, a population-based analysis of VTE rates and associated risk factors showed that 22% of cases were associated with malignancy (18) and is comparable with the rate reported in a UK population (18.6% of all VTEs) (72).

The hospital-based studies reported rates ranging from 0.5 to 22.4%. In contrast to the population-based studies, these Asian hospital-based incidence rates of CAT seem to be at least comparable to those reported outside Asia, where the frequency of VTE in cancer patients admitted to the hospital ranges from 2 to 12% (72).

Among the hospital series, only two studies, one from India and one from Japan, reported CAT rates in all patients regardless of cancer site. However, the study from India, which reported a very low rate of CAT (0.5%) (24), included predominantly symptomatic VTE only. Furthermore, the study was on newly diagnosed cancer patients, which generally have a lower prevalence of VTE compared to patients with advanced disease or those who have received prior chemotherapy. Hence, the low rate reported in this study is not surprising and likely severely underestimates the rate of CAT. On the other hand, the study from Japan, a retrospective review of patients with cancer, reported a 13% rate of DVT or PE (25), which still approximates the rates reported in Asian and Western population-based studies.

The possible reasons for the higher CAT rates in hospitals are varied and would include higher-risk and potentially sicker patients being incorporated in the hospital-based studies. However, this also supports the possibility of underdiagnosis of CAT among patients seen in outpatient clinics. Hospital data alone are likely to be insufficient to estimate incidence rates of VTE in patients with cancer, and rates may vary if studies do not include primary care, cause of death and autopsy data.

### Sites of VTE

VTE in unusual sites are more commonly associated with cancers but literature on the proportions of CAT occurring at unusual sites among cancer patients outside of Asia is limited. Among the Asian population-based and hospital series that reported CAT of all sites, high proportions of unusual sites of thrombosis were reported (in some instances close to 50%) (16, 31, 38, 39). Most of these unusual sites VTEs were picked up incidentally during staging imaging. In contrast, one cohort study in the Netherlands found that VTE in unusual sites contributed only around 7% of all CAT (73).

Consistent with a UK population-based study (72), the population-based study from Taiwan also reported higher rates of PE than extremities DVT (16).

Given the paucity of Asian data regarding sites of VTE in CAT, the real rate of VTE at various sites remain unclear. Future well-controlled studies are required to evaluate this seemingly higher proportion of CAT at unusual sites observed in Asia and to ascertain if the differences, if indeed present, are related to the biological predilection of CAT to sites other than lower limb and pulmonary vasculatures amongst Asian patients or are entirely due to variations in study methodology.

### Cancer Types and Cancer Sites

In Western literature, the cancer sites most associated with thrombosis are the pancreas, stomach, brain, lung, kidneys, uterus, and ovaries, as well as myeloproliferative or myelodysplastic disorders (74) whereas cancers of squamous cell origin have lower CAT rates (15). In this review, similar trends were noted, with gastrointestinal cancers, lung cancer, urogenital/gynecological cancer and hematological cancers being among those with the highest rates of DVT (16, 18, 19, 40). Unlike in Western literature, this review noted that pancreatic cancer was not associated with the highest thrombotic risk in Asia.

The thrombogenicity of various cancer types most associated with CAT was generally consistent between the population-based studies and the hospital-based studies.

Similar to the West, metastatic cancer is associated with the highest rates of VTE, and suggests that advanced cancers are a highly prothrombotic condition that negates the protective effects seen in Asians. Importantly, recurrent VTE can be considered a marker of the biological aggressiveness of the disease, which results in earlier death (22).

### Impact of VTE in Cancer Patients: Morbidity, Mortality, and Recurrences

VTE is known to increase mortality in patients with cancer. In our review of population data, the mortality rates of cancer patients with VTE were slightly more than twice that of cancer patients without VTE (15, 23, 39). Amongst VTE patients, those with recurrent VTE had shorter overall survival than those with non-recurrent VTE (22). Although this mortality impact is important, the effect of VTE on mortality in Asia seemed less than in the West, where risk ratios for mortality rates for cancer-associated VTE compared to those without VTE were reported to be 3.0 and 3.8 in Denmark (8) and Norway (75), respectively, and 6.4 from the RIETE registry (76) with a large number of patients from Europe and Americas.

The reported mortality rates for cancer patients with thrombosis varied widely, from 7.5 to 78.3% in the hospital-based studies. As these studies are very heterogeneous in methodologies and study cohorts, comparisons across studies are not feasible. As with Western studies, this review found that VTE-related deaths (range 0.7–4.2%) were mostly associated with PE and mostly from hospital-based studies (27–29, 39). However, the interpretation of mortality findings was complicated by varying ways of mortality reporting over various timeframes. Nonetheless, our review confirms the substantial impact of CAT on the survival of patients with cancer. Further studies are warranted to address the predictors of death in cancer patients with VTE.

The recurrence rates of VTE in cancer patients are also increased. A retrospective analysis of cancer-associated VTE in patients with advanced solid cancers using data from the Korean VTE registry showed that the 6- and 12-month cumulative incidences of recurrent VTE in cancer patients were 20.6 and 27.0%, respectively. Recurrences within 3 months after index VTE event presented as isolated PE (51.0%), isolated VTE (28%), and PE plus DVT (66%) (22). Pancreas as the primary tumor site, poor performance status and initial presentation with PE are independent risk factors for recurrent VTE in patients with advanced solid tumors receiving anticoagulation therapy after index VTE. Recurrent VTE can be considered a marker of biological aggressiveness of the disease, which results in earlier death (22).

### Treatment and Prophylaxis of VTE

Although we do not specifically evaluate the prophylaxis and treatment of CAT in this current review, several epidemiological studies included provide some interesting data on the use of chemical thromboprophylaxis and anticoagulation treatment of CAT.

In line with current recommendations at the time of publications, LMWH was the main anticoagulant reported although the treatment duration of anticoagulant therapy reported seemed to be also shorter than those recommended in clinical guidelines (16). The two studies that reported warfarin being the main anticoagulant used were conducted in the 2000s (16, 33). In contrast, the landmark CLOT study that defined the role of LMWH as the standard of care in the treatment of CAT (73, 77) was published in 2003. This suggests that in some areas in Asia, the transition to increased use of heparins in the treatment of CAT had been slow.

The use of prophylactic anticoagulation was reported to be generally low. While the rate of CAT in Asia seems to be lower compared to the West, it is not clear whether this could justify the low rate of prophylactic anticoagulation in Asia, especially considering the increasing trend of CAT prevalence in the region. Of interest, Lee et al., (30) in a prospective hospital-based study, reported thromboprophylaxis rate of <5% in patients with gastric cancer undergoing major operation yet their CAT rate was below 1%. Although more studies are needed to validate these remarkable findings, this seemed to suggest that routine peri-operative thromboprophylaxis advocated based on predominantly Caucasian data might not benefit Asian patients to the similar extent. On the other hand, Song et al. (64) reported a VTE rate of 36.8% in patients with lung cancer undergoing lung surgery who did not receive thromboprophylaxis; and reflects the true incidence of VTE in the natural state. This high rate underscores the need for thromboprophylaxis among high-risk patients among Asian patients. There is a need to improve on risk stratification to identify patients who would benefit from thrombo-prophylaxis in CAT.

The risk of bleeding is always an important consideration in the use of thromboprophylaxis. Although bleeding risk is not a predefined objective of our systemic review, we noted most, if not all, of the papers included in this review did not study and report the bleeding rates of included patients. There is also a scarcity of published data comparing the bleeding risk between Asian and Caucasian patients with cancer. Therefore, we recognize that there is an urgent need to evaluate the bleeding risk of Asian cancer population to better inform the clinicians of the overall benefit/risk ratio of thromboprophylaxis.

Accessibility to prophylactic anticoagulation is another factor to improve thromboprophylaxis rates. For example, reimbursement for outpatient use of LMWH by the National Health Insurance of Taiwan was limited to pregnant patients with prosthetic valve replacement (15). While guideline-directed management of patients with CAT would entail prophylaxis, compliance with this recommendation should be augmented with access measures such as medical reimbursements.

### CAT Risk Factors and CAT Prediction

In a population-based study, Chew et al. (16) found specific cancer sites, prior history of VTE, arterial embolism, hypertension, obesity, major surgery, chemotherapy, and combination therapy as risk factors for VTE. Few hospital series included in this review evaluated additional CAT risk factors in multivariate models and some common, non-cancer specific, risk factors identified are advanced age, chemotherapy and poor performance status. These risk factors are also common VTE risk factors and no additional CAT risk factor specific to Asian populations was noted.

Known Risk Assessment Scores (RAMS) have not been widely validated in Asia. While the Caprini score was effective in predicting VTE in a cohort of Chinese patients with lung cancer undergoing lung surgery (64), the Padua score required modification in using the Asian BMI for obesity, and addition of other risk factors such as female gender and hospitalization days for it to be effective in predicting the occurrence of CAT in a small cohort of Korean patients (64). The Ottawa score, a clinical prediction rule for recurrent venous thromboembolism (VTE) in cancer patients was not relevant when applied to a cohort of 546 Korean patients with cancer. This was attributed to the differences between the derivation and validation cohorts in that the Korean cohort had twice the proportion of high risks gastro-intestinal cancers and that the Korean patients with VTE were mostly treated with vitamin K antagonists, which is associated with a higher recurrence rate, rather than LMWH.

In contrast, Yu et al. (15), used the Taiwanese National Health Insurance database to develop a unique scoring system specifically for Taiwanese patients. The developed scoring system was later validated in a second cohort as being effective in predicting the risk for VTE. The scoring system had the highest utility in discriminating those with high risk, which had a 23-fold increased risk of VTE. While the sensitivity and positive predictive values of this scoring system is a reflection of the generally low incidence of VTE in the Taiwanese population, the high NPV is useful in identifying the groups of low-risk patients who will not benefit from pharmaco-prophylaxis.

The patient cohort of this Taiwanese study was heterogenous, including patients of all cancer types, with and without chemotherapy (15). Information on cancer stage, details of therapy, and biomarkers as risk factors of VTE were not available. It is clearly different from the patient cohort where the more established Khorana score was developed (78). For the Khorana score, a more homogenous group of study subjects comprising mostly of adult neutropenic cancer patients with breast cancer, lung cancer, colon and lymphoma, with data on cancer type, body mass index, and biomarkers including leukocyte count, hemoglobin and platelet levels, were incorporated to form a predictive model to stratify cancer patients into three groups with regard to their risk of VTE score. Hence, risk scores like the Khorana score may not apply to the group of frail Asian cancer patients who did not receive chemotherapy.

This simplified predictive model from Taiwan covered a more generalized cancer population and demonstrated the utility of large-scale population-based data in providing valuable evidence to localize the care of patients with cancer (15). It may be useful in decision-making for adopting thromboprophylaxis in cancer patients, particularly in identifying the low-risk patients who will not benefit from thromboprophylaxis and should not be exposed to unnecessary bleeding risks associated with antithrombotic medications.

More validation studies with these RAMs in Asia are required to identify their potential uses in identifying patients who may benefit from thromboprophylaxis. The limited data on RAMS in Asia showed that such scores will be less applicable if the study populations differ from the derivation cohorts. There is a need to develop new risk scores and to derive applicable RAMS based on local patients, which will be more applicable if validated in other Asian patient cohorts with similar characteristics. More studies are also needed to establish the safety and efficacy of thromboprophylaxis in patients at high risk of developing VTE based on such RAMs.

## Conclusions

The incidence of CAT in Asia is several-fold higher than in the general population with rates substantially higher among hospitalized patients. These Asian figures were, however, substantially lower than those reported in Western populations. However, large prospective multinational epidemiological studies are lacking in Asia, which renders the true incidence of CAT uncertain. Nonetheless, VTE is a distinct disease burden for cancer patients in Asia. The cancer sites most associated with VTE as well as risk factors were similar between Asian and Western studies. As in the West, CAT has a major impact on the survival of patients with cancer. Despite this, only around a tenth of patients receive thromboprophylaxis, with anticoagulation mostly being used to treat, rather than prevent, CAT. Finally, the heterogeneity of studies underscores the importance of conducting well-designed population-based studies and registries to standardise data generation. The development and validation of risk scores to predict recurrent VTE risk will also be useful in practice. Such studies would help establish the burden of VTE among cancer patients and the true relationship between cancer and thrombosis in the overall population in Asia.

## Data Availability Statement

The original contributions generated for the study are included in the article, further inquiries can be directed to the corresponding author.

## Author Contributions

All the authors contributed to the design of the study, collection, analyses, and interpretation of data, writing of the manuscript, and the decision to publish the results.

## Conflict of Interest

LHL has received speaker honorarium from Sanofi and has participated in advisory boards for Bayer and Pfizer. The remaining authors declare that the research was conducted in the absence of any commercial or financial relationships that could be construed as a potential conflict of interest. The funders had no role in the design of the study; in the collection, analyses, or interpretation of data; in the writing of the manuscript, or in the decision to publish the results.
